# Interspecific Semantic Alarm Call Recognition in the Solitary Sahamalaza Sportive Lemur, *Lepilemur sahamalazensis*

**DOI:** 10.1371/journal.pone.0067397

**Published:** 2013-06-25

**Authors:** Melanie Seiler, Christoph Schwitzer, Marco Gamba, Marc W. Holderied

**Affiliations:** 1 Bristol Conservation and Science Foundation, c/o Bristol Zoo Gardens, Bristol, United Kingdom; 2 School of Biological Sciences, University of Bristol, Bristol, United Kingdom; 3 Department of Life Sciences and Systems, University of Torino, Torino, Italy; Texas A&M University, United States of America

## Abstract

As alarm calls indicate the presence of predators, the correct interpretation of alarm calls, including those of other species, is essential for predator avoidance. Conversely, communication calls of other species might indicate the perceived absence of a predator and hence allow a reduction in vigilance. This “eavesdropping” was demonstrated in birds and mammals, including lemur species. Interspecific communication between taxonomic groups has so far been reported in some reptiles and mammals, including three primate species. So far, neither semantic nor interspecific communication has been tested in a solitary and nocturnal lemur species. The aim of this study was to investigate if the nocturnal and solitary Sahamalaza sportive lemur, *Lepilemur sahamalazensis,* is able to access semantic information of sympatric species. During the day, this species faces the risk of falling prey to aerial and terrestrial predators and therefore shows high levels of vigilance. We presented alarm calls of the crested coua, the Madagascar magpie-robin and aerial, terrestrial and agitation alarm calls of the blue-eyed black lemur to 19 individual Sahamalaza sportive lemurs resting in tree holes. Songs of both bird species’ and contact calls of the blue-eyed black lemur were used as a control. After alarm calls of crested coua, Madagascar magpie-robin and aerial alarm of the blue-eyed black lemur, the lemurs scanned up and their vigilance increased significantly. After presentation of terrestrial alarm and agitation calls of the blue-eyed black lemur, the animals did not show significant changes in scanning direction or in the duration of vigilance. Sportive lemur vigilance decreased after playbacks of songs of the bird species and contact calls of blue-eyed black lemurs. Our results indicate that the Sahamalaza sportive lemur is capable of using information on predator presence as well as predator type of different sympatric species, using their referential signals to detect predators early, and that the lemurs’ reactions are based on experience and learning.

## Introduction

The avoidance of predators strongly governs the behaviour of potential prey animals [1]. Many birds and mammals are now known to use alarm call systems with referential and/or urgency signalling. As anti-predator behaviour is usually costly, prey animals might benefit from “eavesdropping” on other species’ alarm calls [1] through early recognition of predator presence. This eavesdropping was demonstrated for birds [2], [3], [4], [5], [6], [7], marmots and squirrels [5], [8], [9], [10], [11], [12]. White-browed scrubwrens (*Sericornis frontalis*) and superb fairy-wrens (*Malurus cyaneus*) flee in response to each other’s aerial alarm calls [2]. Shriner [12] showed that yellow-bellied marmots (*Marmota flaviventris*) and golden-mantled ground squirrels (*Spermophilus lateralis*) responded in the same way to conspecific as to heterospecific alarm calls. In primates, Zuberbühler [13] demonstrated that Diana and Campbell’s monkeys (*Cercopithecus diana* and *C. campbelli*) respond appropriately to each other’s leopard and eagle alarm calls. Interspecific alarm call recognition has also been demonstrated in sympatric ringtailed lemurs (*Lemur catta*) and Verreaux’s sifakas (*Propithecus verreauxi*) [14], [15]. Red-fronted lemurs (*Eulemur rufifrons*) and Verreaux’s sifakas (*P. verreauxi*) have an understanding of each other’s aerial as well as general alarm calls [9].

Interspecific communication between taxonomic groups has so far been reported in a number of mammalian and reptilian species that respond to bird alarm calls [1], Guntheŕs dik-diks (*Madoqua guentheri*) [16], red squirrels (*Sciurus vulgaris*) [17], Galapagos marine iguanas (*Amblyrhynchus cristatus*) [18]. Yellow-casqued hornbills (*Ceratogymna elata*) can distinguish between the leopard and eagle alarm calls of Diana monkeys [19], and four different ungulate species (impala (*Aepyceros melampus*), tsessebe (*Damaliscus lunatus*), zebra (*Equus burchelli*), wildebeest (*Connochaetes taurinus*)) distinguish baboon (*Papio hamadryas ursinus)* alarm calls from other loud baboon calls [20]. In primates, Vervet monkeys (*Cercopithecus aethiops*) react differently to the alarm calls produced by superb starlings in response to raptors as opposed to terrestrial predators [21], [22]. Bonnet macaques (*Macaca radiata*) correctly classified the alarm calls of sambar deer (*Cervus unicolor*) and sympatric Nilgiri langurs (*Trachypithecus johnii*) and Hanuman langurs (*Semnopithecus entellus*) [11].

Generally, signal recognition between species might be based on the convergence of acoustically similar signal attributes [23], [24], [25], [26], [27], [28], [29], or it might be learned [5], [7], [16], [18], [30]. Alarm calls in primates for example are usually short with abrupt onsets and broadband noisy spectra [31]. The same basic alarm call structure is seen in a range of other mammals and birds [32], [33], [34].

To date, neither semantic nor interspecific communication have been tested in a solitary and nocturnal primate species, even though one-third of all primate species are nocturnal and small-bodied, and face a high predation risk mainly due to their small size and their different activity period in comparison to most predators [35], [36], [37], [38], [39]. The behaviour of lemurs, including many nocturnal species, may include strong elements of avoidance of predators such as the harrier hawk (*Polyboroides radiatus*), Madagascar buzzard (*Buteo brachypterus*), fossa (*Cryptoprocta ferox*), or Madagascar tree boa (*Boa manditra*) [40], [41], [42], [43], [44], [45], [46], [47], [48]. It is suggested that group-living lemurs that forage together should be less vulnerable to predators than those foraging in pairs or solitarily because of the benefits of group predator detection [49]. However, solitary-living species cannot profit from group benefits on predator avoidance [49], neither during activity nor during resting periods [50], [51]. Accordingly, solitary, nocturnal and small-bodied lemurs should be particularly vulnerable to predators [49] and therefore should have developed correspondingly more efficient behavioural strategies to avoid and/or detect predators early. Eavesdropping on other species’ alarm calls might therefore be a particularly efficient tool to increase the chance of survival for such solitary species.

Due to the diversity of their social systems (solitary, dispersed pairs, harems), their occurrence in sometimes high densities, and their exposed resting position, the sportive lemurs (*Lepilemur* spp.) of Madagascar lend themselves very well to studying anti-predator strategies of nocturnal prosimians, yet so far they have received notably little scientific attention [52], [53], [54], [55]. Anti-predator behaviour has only been studied in one pair-living *Lepilemur* species (*L. ruficaudatus* [56]), which distinguished between different predator types, increased vigilance and usually showed predator-specific flight responses. Studies on how solitary-living sportive lemurs respond to high predation pressure during the day have as yet not been carried out.

Here we investigate the diurnal anti-predator behaviour of the Sahamalaza sportive lemur, *Lepilemur sahamalazensis*, from northwestern Madagascar, using the species as a model for a solitary-living nocturnal prosimian. Since it received species status, the Sahamalaza sportive lemur has been included on the list of the World's Top 25 Most Endangered Primates 2006–2008 [57] and was recently listed as Critically Endangered by the IUCN (C. Schwitzer, pers. comm.).

During daylight hours the Sahamalaza sportive lemur rests alone in tree holes or in tree tangles [58]. Individuals resting in tree holes usually sit at the entrance rather than inside the hole, possibly to increase sun exposure [58]. Therefore, they are easily accessible for predators like the Madagascar harrier hawk (*P. radiatus*), the fossa (*C. ferox*), and possibly the Madagascar tree boa (*B. manditra*), as well as poachers, as all these predators hunt during sportive lemur resting periods. During our own diurnal observations, 5–14% of the observed individualś behaviors were considered active, usually including a high proportion of vigilance [58]. In a previous playback-experiment we played vocalisations of fossa and harrier hawk to the lemurs and used contact calls of crested coua as a control [59]. About 80% of individuals scanned the sky immediately after playback of harrier hawk calls, and the ground or trees after fossa calls. After both call types, the lemurs’ vigilance increased significantly. Interestingly, after playback of crested coua calls, the animals were less vigilant than before, suggesting that the sportive lemurs have an understanding of the semantic meaning of the contact call of this sympatric living bird species [59]. Based on these observations, we hypothesised that this species might be able to deduce information on predator presence as well as predator type from vocalisations of different surrounding species. Thus in the present experimental field study, we tested if the Sahamalaza sportive lemur was able to distinguish and react appropriately to alarm calls and songs of two sympatric bird species and to the alarm calls towards different predator types and contact calls of a sympatric lemur species. We predicted them to increase vigilance after playbacks of alarm calls of crested coua (*C. cristata*) and Madagascar magpie-robin (*Copsychus albospecularis*) as well as three types of alarm call of blue-eyed black lemur (*E. flavifrons*). We predicted that the animals’ vigilance would not change or even decrease after songs of both bird species and contact calls of *Eulemur*. In direct response to alarm calls of the two bird species, we expected the tested sportive lemurs to immediately change their scanning direction and to scan either up or down, as the birds alarm calls might signal for different kinds of predators. Furthermore, we predicted individual sportive lemurs to distinguish between different types of blue-eyed black lemur alarm calls, and thus to look up in direct response towards aerial alarm calls and down in direct response to terrestrial alarm calls of the blue-eyed black lemur. After agitation calls of the blue-eyed black lemur, we predicted the sportive lemurs to either look up or down, as this call type might signal for various kinds of disturbance. We did not expect any change in scanning direction as an immediate response after presentation of the songs of the two bird species or contact calls of the blue-eyed black lemur.

We furthermore hypothesised that any adequate responses of this solitary sportive lemur species to heterospecific calls are based on learning rather than similarity of calls, and predicted that animals would react to alarm calls and songs of sympatric species according to their meaning rather than their acoustic structure.

## Materials and Methods

### Ethics Statement

This study was conducted under permit from the Madagascan Ministere de l’environnement et des forets (Autorisation de recherche #231/11/MEF/SG/DGF/DCB.SAP/SCB) and was ethically approved by the Welfare & Research Advisory Board of the Bristol, Clifton and West of England Zoological Society.

### Study Site

**Figure 1:**
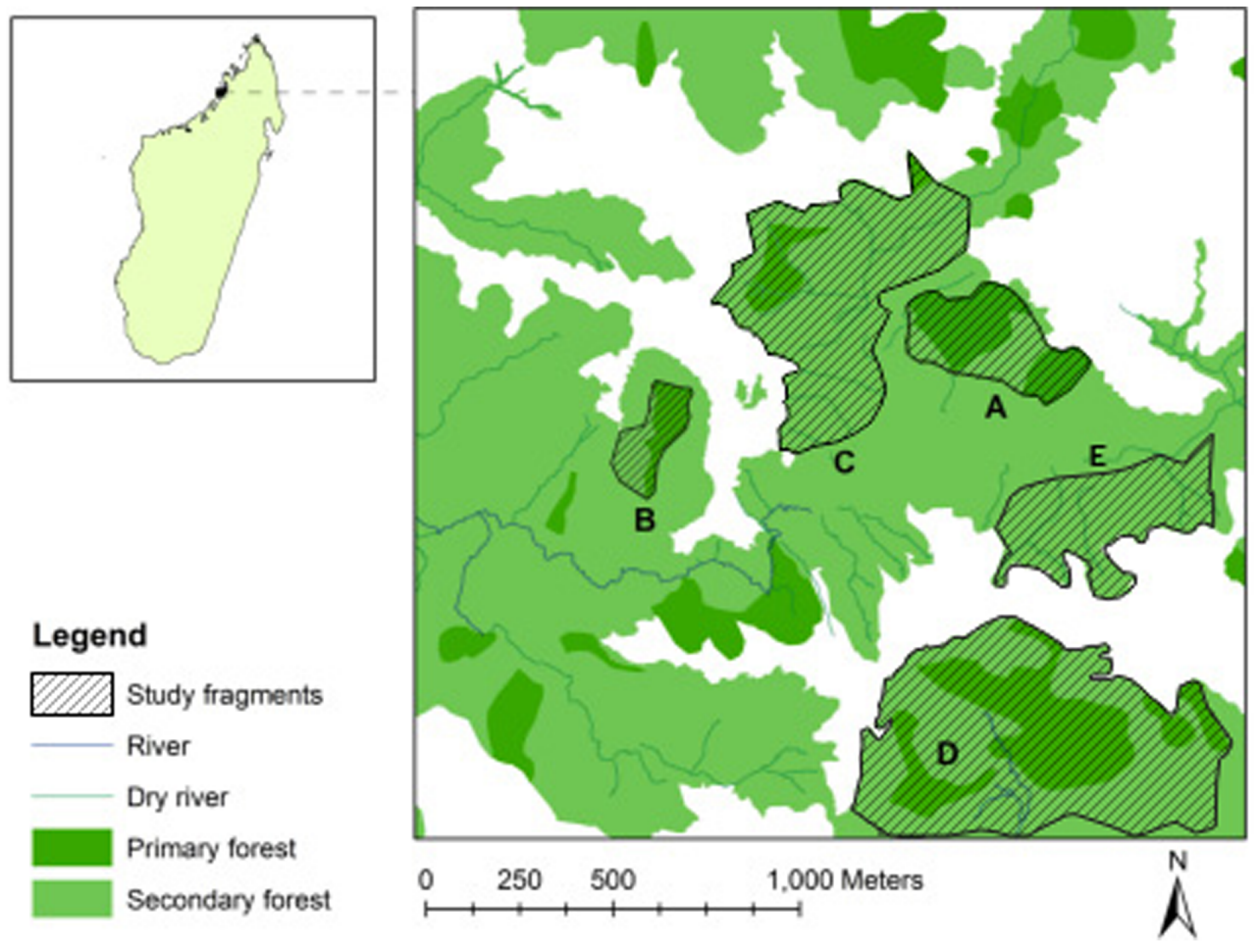
Study fragments (A–E) in the Ankarafa Forest, Sahamalaza Peninsula, Northwest Madagascar.

The Ankarafa Forest is situated in the UNESCO Biosphere Reserve and National Park on the Sahamalaza Peninsula and is part of the Province Autonome de Mahajanga, NW Madagascar. It extends between 13°52′S and 14°27′S and 45°38′E and 47°46′E (WCS/DEC 2002; Figure 1). The climate is strongly seasonal, with a cool, dry season from May to October and a hot, rainy season from November to April. The Ankarafa Forest lies within a transition zone between the Sambirano region in the North and the western dry deciduous forest region in the South, harbouring semi-humid forests with tree heights of up to 30 m [60]. The forests in this area include a mixture of plant species typical of the western dry deciduous forest as well as some typical of the Sambirano domain [61] and comprise primary and secondary forest fragments.

There are no large connected areas of intact primary forest left on the Sahamalaza Peninsula, and the remaining fragments all show some degree of anthropogenic disturbance and/or edge effects [62], [63]. The forests and forest fragments are separated by grassland with shrubs. The Sahamalaza sportive lemur has been confirmed to occur exclusively in this area. Other lemur species in Sahamalaza include the blue-eyed black lemur (*Eulemur flavifrons*), the aye-aye (*Daubentonia madagascariensis*), the western bamboo lemur (*Hapalemur occidentalis*), the northern giant mouse lemur (*Mirza zaza*) and the fat-tailed dwarf lemur (*Cheirogaleus medius*). All lemur species living in Sahamalaza are threatened by hunting and forest destruction [60]. The Ankarafa Forest is home to the Ankarafa research station, where previous research efforts in the region have taken place and which was also the research base for this study.

### Study Subjects

Between September and November 2011, a total of 981 playback experiments were conducted on 19 individual sportive lemurs. The tested lemurs rested at the entrance of tree holes during the day and inhabited five different forest fragments.

The first week of the field season was used to walk the five different forest fragments during the day to find mature sportive lemurs in their resting sites, and to identify them individually by their facial masks where possible. We only chose individuals whose resting site/resting position allowed us to clearly see their faces and thus to observe their behaviour in response to the playback experiments. During this first survey, we found nine individuals. As this *Lepilemur* species is not very abundant and to compensate for the fact that some individuals occasionally changed sleeping sites or disappeared, we also conducted playback experiments on ten additional mature sportive lemurs that we found later in the field season. Due to differences in site fidelity we were not able to play all predator or control calls to all individuals, therefore numbers of sportive lemurs tested in the different categories differ (N = 19 for crested coua alarm and song, Madagascar magpie-robin alarm; N = 18 for magpie robin song and blue-eyed black lemur agitation and contact call). Most sportive lemurs were resting at the entrance of tree holes in dead specimens of *Bridelia pervilleana* at a height of 4.89 (2.24–6.4) m (median with interquartile range). All animals in this study used their sleeping sites on their own. As the study animals were not captured, we are not able to provide information on their sex, size or body mass.

### Playback Stimuli

The alarm vocalisations of two abundant bird species, the Madagascar magpie-robin and the crested coua, were played to the sportive lemurs. Songs of both species were used as control. All calls were obtained from the online archive of the Macaulay Library (http://macaulaylibrary.org). The recordings used for playback procedures are natural call sequences that have been equipped with a 5 second fade in and fade out and were normalised with Avisoft SASLAB Pro (Berlin, Germany). Additionally we played three different types of alarm calls of the blue-eyed black lemur (*E. flavifrons*), i.e. terrestrial, aerial and agitation alarm calls, using the contact calls of the species as control. All calls of the blue-eyed black lemur were recorded in captivity at the Mulhouse Zoo (France), Apeldoorn Apenheul (The Netherlands), or Parco Natura Viva (Italy) by Marco Gamba. Recordings consisted of a single-unit alarm and contact calls, and of agitation calls that consisted of two units [64]. Behavioural observations were associated to each vocalisation [65]: aerial alarm calls were given when large birds flew over the cages, terrestrial alarm calls were uttered when small animals were seen moving on the ground or in the shrubs around the cages, contact calls were emitted while animals were grooming or moving across the enclosure, and agitation calls were emitted when lemurs were jumping around the cage or they were excited (e.g. waiting for food) (Gamba, unpublished data). All recordings have been normalized to match amplitude using Avisoft SASLAB Pro (Berlin, Germany).

**Figure 2:**
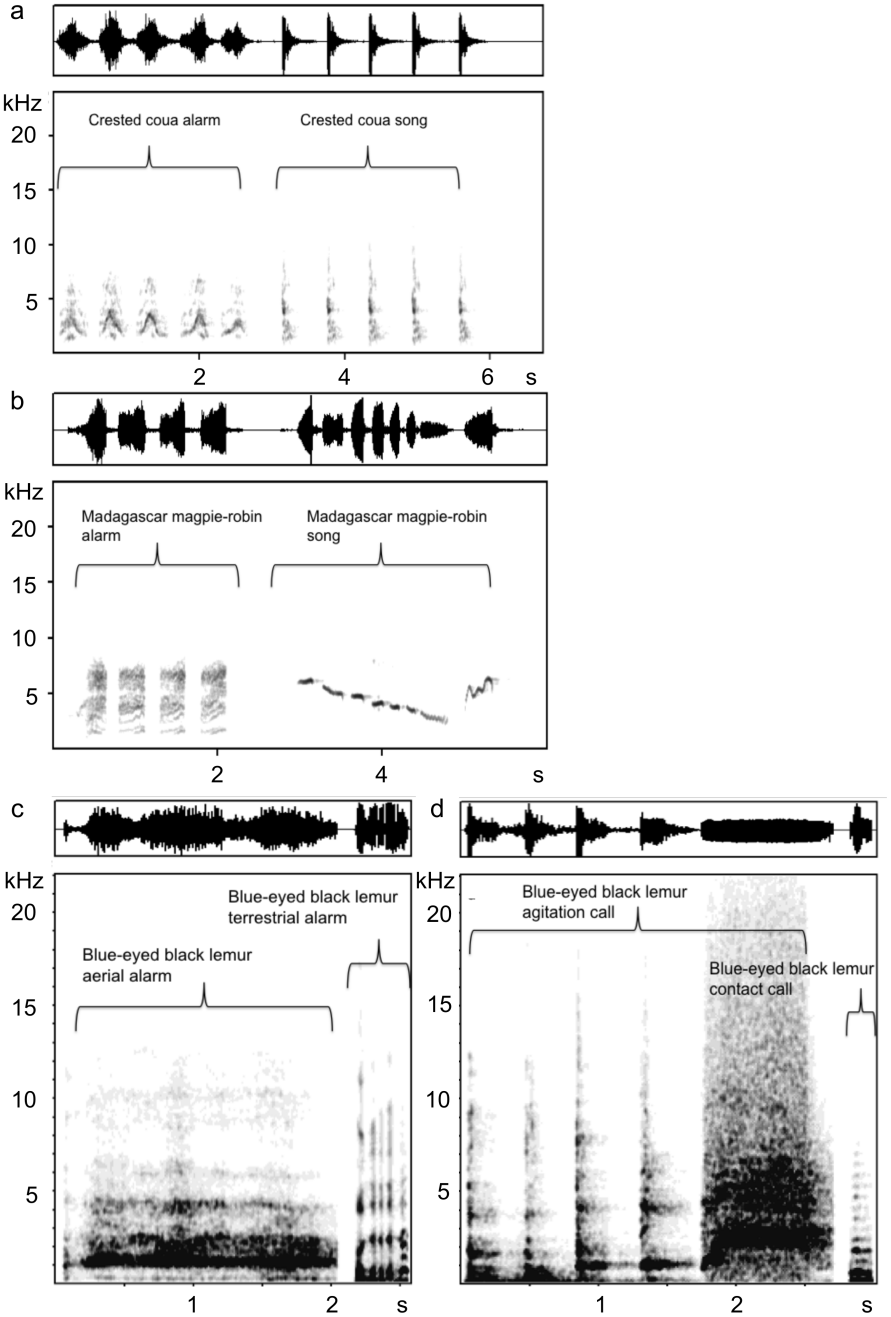
Spectrograms (lower panel) and wave forms (upper panel) of alarm and song of crested coua (a), alarm and song of Madagascar magpie-robin (b) and aerial alarm, terrestrial alarm, agitation call and contact call of blue-eyed black lemur (c,d), used as playback stimuli.

Spectrograms (Figure 2) were generated in Avisoft SASLAB Pro (Berlin, Germany) (1024-point FFT, Hamming window, 48 kHz sampling rate with 0% window overlap resulting in a 47 Hz frequency resolution, and 10.7 ms temporal resolution). To make the experiments replicable and statistically independent, we used songs and alarm calls of five different crested couas, alarm calls of five different Madagascar magpie-robins, songs of four different Madagascar magpie-robins as well as contact calls, aerial, terrestrial and agitation alarm calls of four different blue-eyed black lemurs (Table 1; Figure 2).

**Table 1:** Playback call measurements.

Call type	N	stimulus length (s)	call duration (s)	inter call interval (s)	mean peak frequency (Hz)	source level (dB peSPL)
Crested coua song	5	4.5 (3.3–4.9)	0.2 (0.2–0.3)	0.6 (0.6–0.7)	1870 (1680–2250)	70.4 (70.1–74.7)
Crested coua alarm	5	6.5 (5.8–8.1)	0.5 (0.4–0.6)	0.7 (0.6–1)	1915 (1780–2688)	70.7 (70.7–72.1)
Madagascar magpie-robin song	4	9.5 (8.3–11)	1.8 (1.5–2)	5 (3.6–5.8)	3885 (3750–4613)	71.2 (70.8–71.8)
Madagascar magpie-robin alarm	4	8.7 (4.6–9.9)	0.4 (0.3–0.8)	1.8 (0.5–2.9)	6460 (5810–6650)	71.2 (71.2–71.5)
Blue-eyed black lemur contact call	4	0.1 (0.1–0.1)	0.1 (0.1–0.1)	N/A	680 (660–832.5)	69.6 (69–69.8)
Blue-eyed black lemur aerial alarm	4	1.8 (1.7–1.9)	1.8 (1.7–1.9)	N/A	1200 (1200)	69.9 (69.7–70.3)
Blue-eyed black lemur terrestrial alarm	4	0.4 (0.4–0.5)	0.4 (0.4–0.5)	N/A	250 (230–337.5)	70.3 (69.9–70.5)
Blue-eyed black lemur agitation call	4	1.9 (1.8–2.2)	0.3 (0.3–0.7)	0.3 (0.1–0.5)	1030 (880–1110)	70.2 (69.8–70.6)

Median (interquartile range; Q1–Q3) stimulus length (start of first call unit to end of last call unit), call duration (duration from call onset to call offset), inter call interval (time gap between call offset and successive call onset), peak frequency of call (measured from power spectrum), and source level (in dB peSPL re 1 m) of crested coua song, crested coua alarm, Madagascar magpie-robin song, Madagascar magpie-robin alarm, blue-eyed black lemur contact call, blue-eyed black lemur aerial alarm, blue-eyed black lemur terrestrial alarm blue-eyed black lemur agitation call recordings used as playback stimuli.

**Figure 3:**
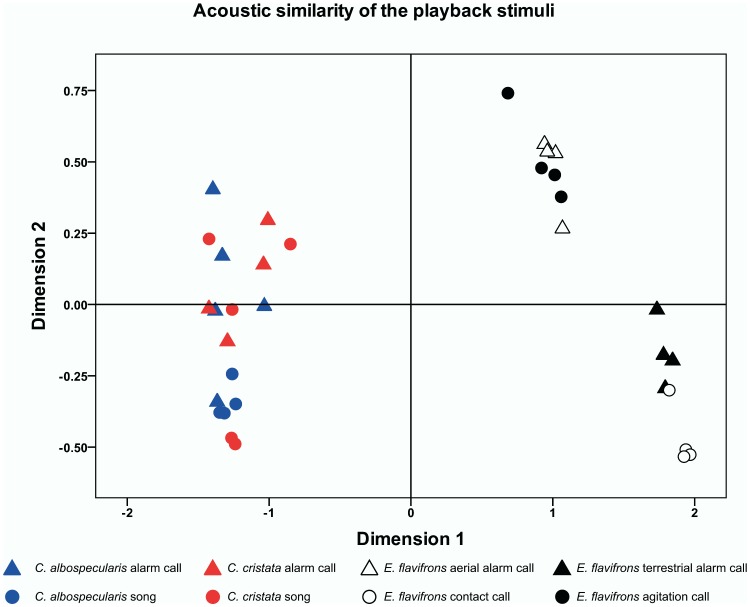
Acoustic distances between alarm calls and songs of crested coua (*C. cristata*) and Madagascar magpie-robin (*C. albispecularis*); and aerial, terrestrial alarm as well as agitation and contact call of blue-eyed black lemurs (*E. flavifrons*); calculated by means of DTWAVE.

To determine the similarity of the calls used as playback stimuli we used an implementation of dynamic time warping [66] available in a freely distributed software package (“DTWave”, http://www.cebl.auckland.ac.nz/DTWave.php). The software uses a sophisticated analysis based on cepstrum coefficients calculation and represents an effective approach to evaluate the similarity of animal vocalisations [66]. Pair wise distances between all of the calls were calculated and organised in a distance matrix (Figure 3). Similarity among calls was then evaluated on the basis of their similarity indices using multidimensional scaling (MDS) in SPSS 20 for Macintosh (IBM SPSS Inc., Armonk, USA).

### Playback Calibration

The calls were played back using an iPod Nano, model A1320 (Apple Inc., Cupertino, CA) and wireless loudspeaker (JBL On Stage Micro II; Harman International Industries, Inc., Stamford, CT; Frequency range 80 Hz-20 kHz). Sound pressure level of call playbacks were measured in a semi-anechoic chamber in Bristol using a 40BF microphone, 26AB preamplifier and 12AA power module (all G.R.A.S. Sound & Vibration, Holte, Denmark) calibrated by D1411E acoustic calibrator (Dawe Instruments, Brentford, UK). Mean sound pressure levels were 69–71 dB peak-equivalent SPL re 1 m (see table 1).

As field test of playback quality we played all stimuli in the absence of lemurs and checked for responses from individuals of the species being played back that were in the vicinity. We obtained vocal responses of crested couas, Madagascar magpie-robins and blue-eyed black lemurs after playbacks of their calls, and sometimes individuals approached us, which confirmed our replays were of adequate quality and level. In cases where we attracted individuals of the replayed species during our experiments with sportive lemurs, the experiment was stopped and that trial was discarded. As far as we are aware, we never elicited responses from predators after playbacks of alarm calls, although we are not able to completely exclude that we might have attracted predators without us noticing them. We did not consider a possible attraction of predators as problematic as alarm calls of both birds and lemurs are commonly heard in the forest fragments.

### Playback Procedure

Playback equipment was either hidden behind a bush or in a tree (0.5–2 m above ground) at a horizontal distance of approximately 5 m from the *Lepilemur* resting site. The observer was seated at a separate position at least 5 m away from the playback equipment. Occurrence, frequency, and duration of responses (see table 2, Categories I) were documented using focal animal sampling for five minutes before and after each playback. Before starting the five-minute pre-playback observation, we waited for the tested individual to settle to the observer’s presence. Sportive lemurs that are not habituated to human presence are vigilant and constantly stare at the potential predator, but return to their usual behaviour [58] after some minutes if the researcher remains calm and does not further approach the animal. During the five-minute observation intervals, the exact time (mm:ss) of the onset and offset of different behaviour was noted. After five minutes, a pre-selected call was played back using a remote control, and the five minutes post-playback observation was started. Additionally, immediate behavioural responses (within 5 s) to playback were noted (see table 2, Categories II). If the animal was out of sight at the time the selected call should have been presented, the experiment was discarded and the whole set was repeated once the animal was in sight again.

**Table 2:** Diurnal ethogram of the Sahamalaza sportive lemur.

*I – Behavioural categories in the five minutes before and after call playback*	
Rest	Animal sits or lies inactively; eyes closed or open, but without attentive scanning
Vigilance	Animal stops an ongoing behaviour and orients head and eyes toward a specific direction or component of the environment or scans the environment. Eyes are wide open, but slight movement still takes place
Autogrooming	Animal grooms itself; licking or gnawing its fur
Change position	Animal climbs slightly up or down the tree tangle or tree hole (max. 50 cm)
Lick/bite tree	Animal licks the surface of its sleeping tree and/or uses its teeth to gnaw off parts of the surface – often observed in combination with Autogrooming
Out of sight	Animal is out of sight in the tree hole or canopy
***II – Behavioral categories immediately (within 5 s) after call playback***	
Scanning up	Animal is vigilant and looks up into sky or trees
Scanning down	Animal is vigilant and looks down to the ground
No change	Animal continues behaviour displayed before the playback of a specific call type

Diurnal ethogram as observed during playback experiments. Durations (in seconds) of category I behaviours were determined within the five minute intervals before and after each playback. Category II was used to quantify behaviour immediately (within 5 s) after each playback.

Call types were presented in a randomised order to individual sportive lemurs in the time window between 8 am and 6 pm. We aimed to play back all four or five versions of the same call type to an individual before repeating a previously presented call. We presented only one song/contact call plus one alarm call to an individual sportive lemur on a single day, and such a playback session lasted approximately 30 to 50 minutes, depending on the time the individual needed to settle to the observer’s presence. Over a period of two months a mean number of 55 (min: 17, max: 78) playback experiments were conducted with individual sportive lemurs. The five different versions of general alarm calls of crested couas were played back between 26 and 28 times, resulting in a total of 137 playbacks to 19 different individuals (Table 3). We used five songs of different crested couas as a control and presented them a total of 140 times to 19 individual subjects (Table 3). We used the five alarm calls of the Madagascar magpie-robin a total of 147 times (Table 3). The four control calls of the Madagascar magpie-robin were played back a total of 117 times to 18 individuals (Table 3). Of blue-eyed black lemurs we presented the four different aerial alarm calls a total of 113 times, and the four different terrestrial alarm calls 106 times to 19 individuals; and the four different agitation calls 107 times to a total of 18 sportive lemurs (Table 4). The four contact calls of different blue-eyed black lemurs used as control were played back a total of 114 times to 18 sportive lemurs (Table 4).

**Table 3:** Numbers of experiments per individual and playback type for bird vocalisations.

L	Crested coua alarm					Crested coua song					Madagascar magpie-robin alarm					Madagascar magpie-robin song				∑
	1	2	3	4	5	1	2	3	4	5	1	2	3	4	5	1	2	3	4	
1	1	0	1	0	0	1	0	0	1	0	1	1	0	0	0	1	1	0	1	9
2	2	2	2	2	2	2	2	2	2	2	2	2	3	1	2	1	2	2	2	37
3	1	1	1	2	1	2	2	2	1	3	2	3	3	1	2	1	1	1	1	31
4	2	2	2	2	2	2	2	2	2	2	2	2	2	2	2	2	2	2	2	38
5	2	2	2	2	2	2	2	2	2	2	3	2	2	1	2	2	2	2	2	38
6	2	2	2	2	2	2	2	2	2	2	1	2	1	3	2	2	3	3	2	39
7	1	1	1	1	1	2	0	1	1	1	1	1	1	1	1	1	1	1	1	19
8	2	2	2	2	2	2	2	2	2	2	2	2	2	2	2	2	2	2	2	38
9	1	1	0	0	0	1	0	1	0	1	1	1	1	0	1	1	1	1	1	13
10	2	2	2	2	2	2	2	2	2	2	2	2	2	2	2	2	2	2	2	38
11	2	2	2	2	2	2	2	2	1	2	2	2	2	2	2	3	2	2	2	38
12	1	1	2	1	1	1	1	2	1	1	2	2	1	2	1	1	2	2	2	27
13	1	2	2	2	4	2	2	2	2	1	2	2	2	2	2	2	2	3	2	39
14	1	1	1	2	1	1	2	1	1	1	2	2	0	2	2	1	1	2	1	25
15	1	0	0	1	1	1	2	0	1	0	2	1	0	1	0	1	0	1	1	14
16	1	1	1	1	1	1	1	1	1	1	0	1	1	1	1	1	1	1	1	18
17	1	0	0	0	0	1	0	0	0	0	0	1	0	0	0	0	0	0	0	3
18	2	2	2	2	2	2	2	2	2	2	2	3	2	2	2	2	2	2	2	39
19	2	2	2	2	2	2	2	2	2	2	2	2	2	2	2	3	2	2	1	38
∑	28	26	27	28	28	31	28	28	26	27	31	34	27	27	28	29	29	31	28	541

Numbers of playback-experiments conducted with five different versions of crested coua alarm and song, Madagascar magpie-robin alarm, and four version of Madagascar magpie-robin song with each sportive lemur (L).

**Table 4:** Numbers of experiments per individual and playback type for blue-eyed black lemur vocalisations.

L	Aerial alarm				Terrestrial alarm				Agitation call				Contact call				∑
	1	2	3	4	1	2	3	4	1	2	3	4	1	2	3	4	
1	1	0	0	0	0	0	1	1	1	0	1	0	0	0	1	1	7
2	2	1	2	2	2	2	2	2	2	2	2	2	2	2	2	2	31
3	2	1	2	1	1	1	1	1	1	1	1	1	1	1	1	1	18
4	2	2	2	2	2	2	2	2	2	2	2	2	2	2	2	2	32
5	2	2	2	2	2	1	2	2	2	2	3	1	2	2	2	2	31
6	2	3	2	2	3	3	2	2	2	2	1	2	3	2	2	2	35
7	1	0	1	1	0	0	1	0	1	0	0	0	0	2	1	1	9
8	2	2	2	2	2	2	1	2	2	2	2	2	2	2	2	2	31
9	1	0	1	0	1	0	1	0	1	0	0	0	0	1	1	1	8
10	2	2	3	2	2	2	2	2	2	2	2	2	2	2	2	2	33
11	2	2	2	2	2	2	2	2	2	2	2	2	2	2	2	2	32
12	2	1	2	1	1	2	1	1	1	0	1	1	2	2	2	1	21
13	3	2	1	2	2	2	2	2	2	2	2	2	2	2	2	2	32
14	2	1	1	1	2	1	1	1	2	1	2	1	1	2	2	1	22
15	0	1	0	2	1	1	0	0	1	1	1	1	0	1	1	1	12
16	1	1	2	1	1	1	2	1	2	1	2	1	0	1	1	1	19
17	1	0	0	0	0	1	0	0	0	0	0	0	0	0	0	0	2
18	2	2	2	2	2	2	2	2	2	3	2	2	3	2	2	2	34
19	2	2	2	2	2	1	2	2	2	2	2	2	2	2	2	2	31
∑	32	25	29	27	28	26	27	25	30	25	28	24	26	30	30	28	440

Numbers of playback-experiments conducted with four different versions of blue-eyed black lemur aerial and terrestrial alarm calls, agitation and contact call with each sportive lemur (L).

### Data Analyses

To test for differences in the duration of individual lemurs’ vigilance (measured as seconds of vigilance) before and after the playback of predator and control calls, we performed a Wilcoxon signed rank test (P≤0.05) on each individuals’ mean vigilance duration in the five-minute periods before and after the playback of each stimulus type.

To test for immediate responses, scanning directions were either rated as appropriate or inappropriate. We classified scanning up or down after alarm calls of crested coua and Madagascar magpie-robin as appropriate, as these alarm calls might refer to different kinds of predators. The response was also rated as appropriate if individuals scanned the sky after blue-eyed black lemur aerial alarm or if they looked down after blue-eyed black lemur terrestrial alarm calls. We rated scanning up or down after blue-eyed black lemur agitation calls as appropriate, as this call type might refer to different sources of disturbance. Furthermore, no change of scanning direction after songs/contact calls of either species was classified as appropriate behaviour. Consequently we classified no reaction after alarm calls and scanning up or down after songs/contact calls as inappropriate behaviour.

χ^2^ tests with Yates-correction of numbers of appropriate and inappropriate behaviour of each individual were used to test for significant differences in the reactions of lemurs towards the playback stimuli (rate 50%; P≤0.05). χ^2^ tests were also used to test if the sportive lemurs increased or decreased various behaviour displayed immediately before in comparison to immediately after the playbacks.

## Results

### Acoustic Similarity of the Playback Stimuli

We used multidimensional scaling to identify patterns in the distance matrix of acoustic similarity indices generated with DTWAVE (Figure 3). The distances based on acoustic similarity allow identification of three main clusters. The calls of blue-eyed black lemurs are grouped in two different clusters (aerial/agitation calls and contact/terrestrial alarm calls. figure 3), which are clearly separated from each other and from the cluster including alarm calls and songs of crested coua as well as Madagascar magpie-robin. The analysis did not reveal a difference in the distances between songs and alarm calls of both bird species (Figure 3).

### Duration of Vigilance

63% and 68% out of 19 individuals responded with increased duration of vigilance after playbacks of alarm calls of crested coua and Madagascar magpie-robin, whilst only 5% and 15% decreased the duration of vigilance after crested coua and Madagascar magpie-robin alarm calls, respectively. Overall, the duration of vigilance increased after playbacks of crested coua and Madagascar magpie-robin alarms (Table 5). After aerial alarm calls of blue-eyed black lemurs 68% of the individuals increased vigilance, whilst 16% did not change the amount of vigilance. After presentation of the terrestrial alarm call and agitation call, no changes in the overall duration of vigilance were found (Table 5). No individual ever vocalised in response to any of the call replays nor did they ever show a flight response.

**Table 5:** Changes in sportive lemur vigilance in response to playbacks.

Call type	Vigilance before call (s)	Vigilance after call (s)	
Crested coua alarm	104.5 (36.5–166.8)	126 (78–229.3)	P = 0.004
Madagascar magpie-robin alarm	50 (18.8–64.8)	95.5 (65–133.8)	P = 0.003
Blue-eyed black lemur aerial alarm	40 (8.3–68.5)	95 (39–153)	P = 0.011
Blue-eyed black lemur terrestrial alarm	32.5 (7.8–61.5)	40 (18.5–70.3)	P = 0.449
Blue-eyed black lemur agitation call	44.3 (13.1–107.5)	54.3 (40.8–99.5)	P = 0.586
Crested coua song	80.5 (43.8–146.8)	65 (21.5–88.5)	P = 0.01
Madagascar magpie-robin song	50 (16.6–100.9)	39.8 (11.6–72.9)	P = 0.360
Blue-eyed black lemur contact call	54.5 (15.5–63)	18.5 (0–47.4)	P = 0.179

Median (quartile 1– quartile 3) vigilance in seconds within 5 min before and after the playback of alarm calls of crested coua (N = 19), Madagascar magpie-robin (N = 19), blue-eyed black lemur (All: N = 19) and songs of crested coua (N = 19), Madagascar magpie-robin (N = 19) and blue-eyed black lemur contact calls (N = 19). Wilcoxon Signed Ranks Test with α ≤0.05.

After crested coua songs the vigilance of tested individuals decreased (Table 5). After Madagascar magpie-robin song and blue-eyed black lemur contact call, there were no significant changes in the overall duration of vigilance (Table 5).

### Immediate Behavioural Changes

In direct response to crested coua and Madagascar magpie-robin alarm calls, 94% and 89% of the individuals changed their behaviour from resting or autogrooming to vigilance, respectively. Looking at the number of trials, the percentage of vigilance (amount of vigilance in relation to non-vigilant behaviours immediately before and after the playbacks) increased significantly, whilst resting decreased (Table 6). After playbacks of blue-eyed black lemur aerial alarm, 89% of the observed animals changed their behaviour from resting or autogrooming to vigilance in direct response to the stimuli, and the overall vigilance increased significantly (Table 6). The percentages of observed behaviour did not change after the presentation of songs of both bird species and terrestrial alarm, agitation or contact call of blue-eyed black lemurs (Table 6).

**Table 6:** Changes in sportive lemur behaviours (resting, vigilance, autogrooming) in response to playbacks.

	Rest % before	Rest % after	P	Vigilance % before	Vigilance % after	P	Autogrooming % before	Autogrooming % after	P
Crested coua alarm	68 (36.5–100)	22.5 (0–67)	<0.05	27.5 (0–55)	74.5 (33.5–100)	<0.05	4.5 (0–100)	3 (0–20)	>0.1
Crested coua song	61 (0–100)	51 (0–100)	>0.1	31 (0–100)	40 (0–100)	>0.1	8 (0–20)	9 (0–50)	>0.1
Madagascar magpie-robin alarm	68 (30–100)	24 (0–50)	<0.05	28.5 (0–70)	75 (50–100)	<0.05	3.5 (0–25)	1 (0–9)	>0.1
Madagascar magpie-robin song	72 (25–100)	61 (25–100)	>0.1	23 (0–75)	35 (0–75)	>0.1	5 (0–20)	4 (0–25)	>0.1
Blue-eyed black lemur aerial alarm	67.5 (0–100)	23(0–100)	<0.05	24 (0–100)	77 (0–100)	<0.05	8.5 (0–28.5)	0 (0)	>0.1
Blue-eyed black lemur terrestrial alarm	72 (0–100)	62 (0–100)	>0.1	24 (0–50)	36 (0–100)	>0.1	4 (0–100)	3 (0–14)	>0.1
Blue-eyed black lemur agitation call	73.5 (0–100)	55 (0–78)	>0.1	19 (0–50)	41.5 (12.5–100)	>0.1	7.5 (0–75)	2.5 (0–25)	>0.1
Blue-eyed black lemur contact call	78 (44–100)	7 (44–100)	>0.1	19 (0–44.5)	20 (0–50)	>0.1	2.5 (0–12.5)	4 (0–22)	>0.1

Mean (minimum-maximum) percentages of behaviours observed immediately before and after presentation of alarm calls and songs of crested coua and Madagascar magpie robins, as well as aerial, terrestrial alarm, agitation, and contact calls of blue-eyed black lemurs. χ^2^ test (rate 50%; P≤0.05).

### Scanning Direction

No individual looked directly in the direction of the speaker in response to the playback stimuli. In total, 77% and 85% of the individuals displayed more appropriate scanning behaviour after the alarm calls of crested coua and Madagascar magpie-robin (looking up in all cases), as did 81% of the individuals after blue-eyed black lemur aerial alarm calls, resulting in significant changes on group level. Table 7 summarises the overall numbers of appropriate or inappropriate reactions in response to the different call types. After terrestrial alarm, agitation calls of blue-eyed black lemur, tested individuals did not show more appropriate or inappropriate behaviour (Table 7). Tested animals did not show more appropriate or inappropriate behaviour after playbacks of crested coua songs, other than in response to Madagascar magpie-robin songs after which they displayed significantly more appropriate behaviour (no change of scanning direction). In response to the blue-eyed black lemur contact call, the tested individuals usually did not react and therefore showed appropriate behaviour (Table 7).

**Table 7:** Appropriate and inappropriate responses of Sahamalaza sportive lemurs to playbacks.

Call type	Appropriate ∑	Inappropriate ∑	
Crested coua alarm	79	36	P<0.001
Madagascar magpie-robin alarm	83	43	P<0.05
Blue-eyed black lemur aerial alarm	61	30	P<0.05
Blue-eyed black lemur terrestrial alarm	29	50	P>0.1
Blue-eyed black lemur agitation call	42	50	P>0.1
Crested coua song	66	52	P>0.1
Madagascar magpie-robin song	65	23	P<0.001
Blue-eyed black lemur contact call	73	21	P<0.001

Appropriate reactions (scanning up or down after crested coua alarm and Madagascar magpie-robin alarm, scanning the sky after blue-eyed black lemur aerial alarm, down after blue-eyed black lemur terrestrial alarm and either up or down after blue-eyed black lemur agitation call, no change of scanning direction after songs/contact calls of each species) or inappropriate (no reaction after alarm calls; scanning up or down after songs/contact calls) of scanning direction of tested sportive lemurs. χ^2^ test (rate 50%; α≤0.05); Degrees of freedom (Df) = 12 for crested coua alarm, 13 for crested coua song, 12 for Madagascar magpie-robin alarm, 10 for Madagascar magpie-robin song, 11 for blue-eyed black lemur aerial alarm, agitation call and contact call; 9 for blue-eyed black lemur terrestrial alarm.

## Discussion

Our results suggest that the Sahamalaza sportive lemur is capable of taking advantage of other species’ alarm calls. As predicted, tested sportive lemurs significantly increased vigilance after playbacks of alarm calls of sympatric bird species, the crested coua and the Madagascar magpie-robin. Furthermore, they responded with increased vigilance to aerial alarm calls of the sympatric blue-eyed black lemur, but contrary to our prediction, not to their terrestrial alarm and agitation calls.

In response to playbacks of songs/contact calls of the three species, the sportive lemurs became significantly less vigilant after songs of the crested coua, and vigilance also decreased, though not significantly, after songs/contact calls of the Madagascar magpie-robin as well as the blue-eyed black lemur, possibly because these songs/contact calls are indicating that no predator is around. These results show that this *Lepilemur* species is able to distinguish between alarm calls and songs of at least two sympatric bird species as well as between aerial alarm and contact calls of the blue-eyed black lemur.

This kind of interspecific communication between taxonomic group was so far only found in diurnal and group living animals (red squirrels (*Sciurus vulgaris*) [17]; Gunther’s dik-diks (*Madoqua guentheri*) [16]; banded mongooses *(Mungos mungo*) [1]; Galápagos marine iguanas (*Amblyrhynchus cristatus*) [18]; yellow-casqued hornbills (*Ceratogymna elata*) [19]; impala (*Aepyceros melampus*) tsessebe (*Damaliscus lunatus*)*;* zebra (*Equus burchelli*)*;* wildebeest (*Connochaetes taurinus*) [20]; vervet monkeys (*Cercopithecus aethiops*) [21], [22]; Bonnet macaques (*Macaca radiata*) [11]).

In immediate response to alarm calls of the crested coua and the Madagascar magpie-robin the tested animals displayed significantly more appropriate scanning behaviour. Animals usually scanned the sky, indicating that the birdś alarm calls might signal for raptors rather than predators in general. Furthermore, in line with the results on change of duration of vigilance, sportive lemurs displayed significantly more appropriate scanning behaviour after aerial alarm, but not after terrestrial alarm and agitation calls of the blue-eyed black lemur. The tested individuals scanned towards the sky, but never to the ground when they reacted to aerial alarm, suggesting that they expected the potential danger from above and thus classified the alarm call correctly. In conclusion, the tested Sahamalaza sportive lemurs seem to understand the semantic of the aerial alarm call of the blue-eyed black lemur, and change their behaviour accordingly, but it remains unclear if agitation and terrestrial alarm calls are understood and not deemed important or if they are not classified as alarm calls. Agitation calls are usually given in inter- and intragroup encounters and conflicts (M Seiler, pers. obs.), so it might not be sensible for sportive lemurs to react in response to them. In immediate response to the songs of the crested coua and Madagascar magpie-robin, as well as to the contact calls of the blue-eyed black lemur, the tested individuals usually did not react, indicating that they did not associate a possible risk with these stimulus types and thus classified the songs/contact calls correctly.

The recognition of signals between species is either based on the convergence of acoustically similar signal attributes [23], [24], [25], [26], [27], [28], [29], or it is learned [5], [7], [16], [18], [30]. In our experiment, the birds’ alarm calls are usually short with abrupt onsets and broadband noisy spectra, but these characteristics are shared with the song of the crested coua (see figure 2). Nevertheless, sportive lemurs responded differently and adequately to alarm call and song of the crested coua. Similarly, aerial alarm call and agitation call of the blue-eyed black lemurs were perceptually similar to the human ear, but the tested sportive lemurs displayed more vigilance and appropriate behaviour after the call type that indicates the presence of a raptor, suggesting that their responses are based on learning rather than on similarities in signal structure. The learning hypothesis is also confirmed from the acoustic similarity analysis of the playback stimuli. The alarm calls of blue-eyed black lemurs were clearly grouped apart from all birds’ vocalisations, discarding the possibility that acoustic similarity of the alarm vocalisations plays a role in eliciting vigilance and anti-predator behaviour in the sportive lemurs. This is in agreement with studies showing that various animal species respond appropriately to alarm calls that are acoustically different from their own [5], [7], [16], [18], [30], suggesting that their responses had been learned. For example, golden-mantled ground squirrels (*Spermophilus lateralis*) that respond to yellow-bellied marmot (*Marmota flaviventris*) alarm calls [12] could be trained to associate a new sound with the appearance of a model predator [30]. Young vervet monkeys (*Cercopithecus aethiops*) acquired the ability to respond to the alarm calls of superb starlings (*Spreo superbus)* faster when they were exposed to a higher level of starling alarm calls, suggesting learning [67]. Several studies have also demonstrated that species respond to the alarm calls produced by sympatric but not allopatric species [5], [9], [10], [11]. For example, red-fronted lemurs (*Eulemur rufifrons*) and Verreaux’s sifakas (*Propithecus verreauxi*) responded appropriately to each others aerial and general alarm calls, but not to baboon alarm calls [9]. Bonnet macaques (*Macaca radiata*) correctly classified the alarm calls of sambar deer (*Cervus unicolor*), sympatric Nilgiri langurs (*Trachypithecus johnii*) and Hanuman langurs (*Semnopithecus entellus*), and call recognition was highest in adults and in regions where individuals were frequently exposed to the calling species [11]. We therefore suggest that the responses of the Sahamalaza sportive lemur towards alarm calls of the crested coua, Madagascar magpie-robin and the aerial alarm call of the blue-eyed black lemur are based on experience and learning. It is therefore likely that this species also makes use of alarm calls of other sympatric species.

Although the Sahamalaza sportive lemur clearly reacted to the alarm calls of three different sympatric species, their responses were not as strong as when we directly presented vocalisations of an aerial and a terrestrial predator in a previous study [59]. A total of 73% of the individuals scanned the sky immediately after playback of Harrier hawk calls, and 100% of the individuals scanned the ground or trees after fossa calls; after both call types the lemurs’ vigilance increased significantly.

In conclusion, our results in this study suggest that the Sahamalaza sportive lemur is able to increase the chance of detecting a predator early through eavesdropping on sympatric species’ alarm calls, in addition to its predator specific anti-predator behaviours that include early acoustic detection and keeping track of predators. This additional “eavesdropping” might be an essential ability for a solitary living animal, which cannot count on early predator detection through group members. The ability of learning the meaning of other species’ alarm calls is therefore an important factor of the anti-predator behaviour of the Sahamalaza sportive lemur.
